# Integrating Knowledge Translation: A Swiss Approach to Bridging Research and Health System Improvement

**DOI:** 10.1002/lrh2.70056

**Published:** 2025-12-05

**Authors:** Natalie Gray Messerli, Sarah Mantwill

**Affiliations:** ^1^ Competence Center for Learning Health Systems, Faculty of Health Sciences and Medicine University of Lucerne Lucerne Switzerland

**Keywords:** capacity building and co‐creation, evidence‐informed decision making, health system improvement, knowledge translation, learning health system

## Abstract

**Introduction:**

The Swiss Learning Health System (SLHS), funded from 2017 to 2025, facilitated the movement of research to practice and policy, responding to national calls to enhance health services research and build research scientist capacity. This study evaluates the SLHS to understand how Learning Health System (LHS) science can provide a foundation for Knowledge Translation (KT) platforms at a national level, identifying successes, challenges, and lessons learned from capacity building and institutionalizing KT in support of evidence‐informed decision‐making (EIDM) in health policy and practice.

**Methods:**

We employed a mixed‐methods approach from September to December 2023, using the SLHS program aims and the Knowledge‐to‐Action framework to inform the study's conceptualization. Data collection involved two workshops with over 40 SLHS members, a survey of 39 members, and in‐depth interviews with 10 key informants, analyzed using descriptive and thematic methods.

**Results:**

Capacity‐building efforts prompting a cultural shift by training research scientists to adopt a science‐policy‐practice mindset were the most common successes captured. The LHS and KT approaches aided in dismantling silos and encouraged community building through participatory methods. An important lesson learned is the value of co‐creation involving key partners, especially patients, in the research process to strengthen relevance, issue prioritization, and evidence use. However, challenges persisted in adequately tailoring and transferring knowledge, highlighting the need for more consistent engagement with community partners to enhance the impact and relevance of KT efforts.

**Conclusion:**

This study demonstrated that the SLHS is a valued initiative for capacity building, while highlighting the need to strengthen co‐creation and refine strategies for adapting research evidence for EIDM in health policy and practice.

## 1 Introduction

### 1.1 The Swiss Learning Health System

The Swiss Learning Health System (SLHS) was established in 2017 as a national platform to foster dialogue and create learning cycles for continuous health system improvement. Designed to bridge research, policy, and practice, it provided an infrastructure to routinely integrate evidence into decision‐making processes [1]. The SLHS operated until mid‐2025, with several of its activities now continuing under other initiatives.

The SLHS emerged in response to a growing call for health systems to be more adaptive to population needs, emphasizing the role of community partners in shaping and co‐creating solutions. In the early 2010s, publications from the Swiss Federal Office of Public Health (FOPH) and the Swiss Academy of Medical Sciences (SAMS) highlighted the need to prioritize health services research, as challenges related to population aging and a steady transition from communicable to non‐communicable diseases became increasingly pressing [2, 3].

Building on this momentum, and to advance interuniversity collaboration, the SLHS received independent program funding from the State Secretariat for Education, Research, and Innovation (SERI) for the period 2017–2025. Embedded within health services research, the main structural objective of the SLHS was to establish a bridging mechanism between research, policy, and practice, following a Learning Health System (LHS) approach within the Swiss context.

The LHS concept was introduced by the Institute of Medicine (now the National Academy of Medicine) in 2007. Their 2012 definition describes LHS as, “A system in which science, informatics, incentives, and culture are aligned for continuous improvement and innovation, with best practices seamlessly embedded in the care process, patients and families as active participants in all elements, and new knowledge is captured as an integral by‐product of the care experience” [4]. Since its conceptualization, different medical specialties and care models have implemented this framework, as well as different health systems, such as Canada, Denmark, and Switzerland [5].

**FIGURE 1:**
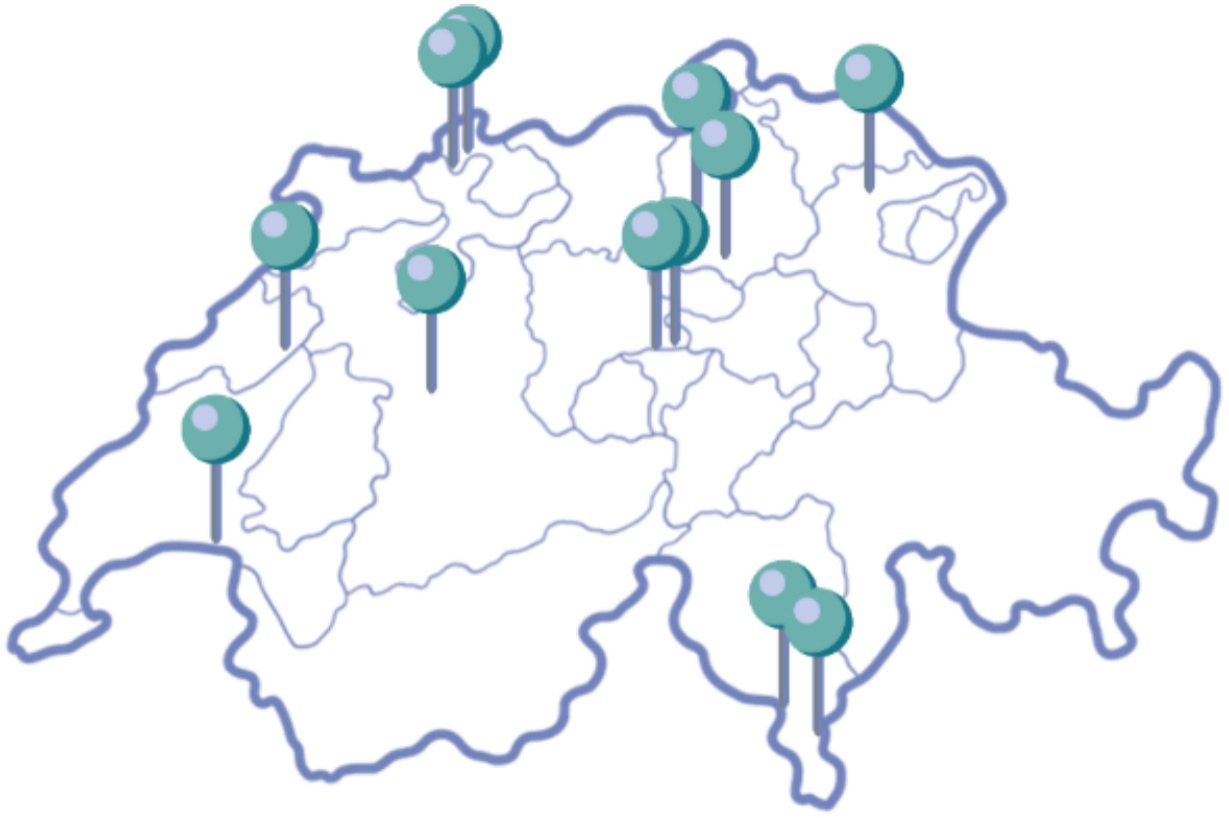
The Swiss Learning Health System's Partner Institutions (2025): Bern University of Applied Sciences, Lucerne University of Applied Sciences and Arts, Swiss Tropical and Public Health Institute, University of Applied Sciences and Arts of Southern Switzerland, University of Basel, University of Italian Switzerland, University of Lausanne, University of Lucerne, University of Neuchatel, University of St. Gallen, University of Zurich, and Zurich University of Applied Sciences.

While traditional LHSs are often implemented within clinical settings, the SLHS was conceptualized as a national‐level system operating at the intersection of academic research, policy, and practice. In its later stages, it brought together a partner network of 12 Swiss institutions of higher education (Figure 1), represented by senior academic professionals on a governing board that provided strategic direction and oversaw key activities, including a research scientist training program [6, 7]. Many of these senior academic professionals were also directly engaged as PhD supervisors and collaborators for the program's doctoral researchers. Administered separately from other federal entities, such as the Federal Office of Public Health (FOPH), the initiative was managed through a dedicated management and coordination office at the University of Lucerne, specifically established to oversee and support the work of the program's consortium. The SLHS received two consecutive rounds of funding for the periods 2017–2021 and 2021–2025. This funding was primarily dedicated to financing doctoral researchers at respective partner institutions, who actively supported specific learning cycles while receiving training in LHS science.

**FIGURE 2:**
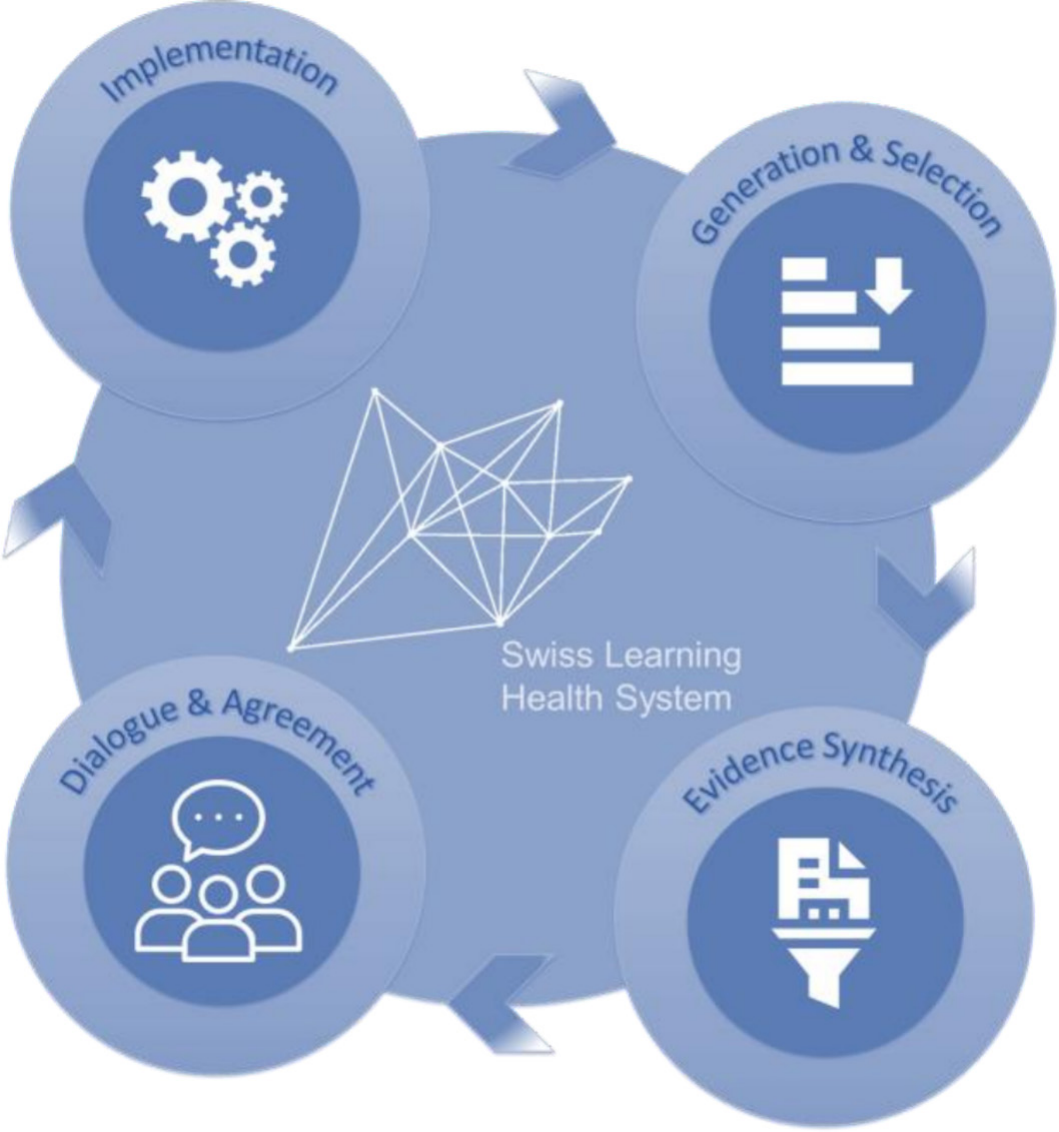
Bridging mechanism in the Swiss Learning Health System (SLHS)—the SLHS learning cycle.

Conceptually, the SLHS established an overarching learning cycle (Figure 2) as a guiding framework for identifying and steering smaller, topic‐specific learning cycles. As a first step, the SLHS learning cycle (SLC) focused on the iterative identification of current or emerging issues within the Swiss health system. This process was informed by the governing board, the health system advisory board, which included representatives from across the healthcare system and individual experts, and by strategies previously developed by federal institutions such as the FOPH.

Once key issues were identified, practitioners and community members with relevant experience were invited to contribute alongside governing board members for each specific issue. For every issue, these initial partners formed an initial learning community, which worked together to further develop the topic into a dedicated SLHS topic. Each topic then led to the initiation of its own cycle within the SLHS framework. These topics spanned diverse areas, including interprofessional collaboration, antibiotic prescribing practices, financing of psychosocial services, and various challenges in primary care.

A central influence on how each SLHS topic evolved was the learning community itself. These learning communities evolved over time and brought together diverse partners, including academic collaborators, policy and practice professionals, patients, and caregivers. The scope of each SLHS topic varied from national to local levels, depending on factors such as data availability, linguistic context, and the priorities set by the learning community. For example, antibiotic prescribing practices were addressed at a national level, while the financing of psychosocial services was examined in a more local context.

Each SLHS topic was addressed through existing and newly developed evidence syntheses, providing a knowledge base to inform the co‐development of targeted recommendations. The evidence syntheses, including Policy Briefs, served as a basis for structured dialogues with diverse participants within each SLC. These dialogues were designed to identify the best course of action and support continuous learning [8]. In line with the SLHS framework, subsequent steps included the development of implementation guidance, which should feed back into the cycle and inform the next round of issue identification [1]. Each SLC actively involved senior academic professionals and doctoral researchers from one or multiple partner institutions, supported by the management and coordination office, with doctoral researchers playing a key role in translating knowledge and supporting the operationalization of the individual SLCs.

## 2 Knowledge Translation and the SLHS

Understanding the unique characteristics of the Swiss health system is essential to situating the role of the SLHS. In contrast to centralized systems in countries like the United Kingdom, Denmark, or Sweden, the Swiss system is highly fragmented [9]. Its health system is characterized by its participatory framework, mixed private social health insurance, and decentralized governance with four national languages across 26 cantons and three linguistic regions, with various public and private community partners playing essential roles [10, 11]. Mannamplackal et al. [12], for example, emphasized the role of the SLHS and learning cycles as a potential mitigation approach to regional disparities due to its federalist governance structure, potentially leading to an ability to produce and apply proven approaches in comparable settings.

Building on this need for a coordinated yet flexible framework, the SLHS positioned Knowledge Translation (KT) activities as core components of its approach, including the development of the above‐mentioned evidence syntheses and the facilitation of structured dialogues. Defined as “the synthesis, dissemination, exchange, and ethically sound application of knowledge to improve health and strengthen the healthcare system” [13], KT often follows an integrated KT (IKT) model described as the co‐production of evidence by researchers and knowledge users or implementers [14]. The SLHS expanded this by involving a range of community partners within each LHS topic—including patients, patient representatives, caregivers, professionals in social services, and actors from the private sector such as insurers—not only in structured dialogues but also in developing, for example, evidence syntheses and refining the outputs of the dialogues. This approach ensured that diverse perspectives informed key phases of the SLCs and fostered the formation of potential learning communities within each LHS topic, creating opportunities to surface and prioritize additional emerging issues. While the overarching SLHS framework was guided by a formal governance structure, including a governing board and health systems advisory board, the individual SLCs did not have a dedicated governance body; instead, their coordination and leadership were adapted to the specific needs and priorities formed by the learning community for each SLHS topic. However, to ensure consistency and quality across initiatives, key steps in the SLC, such as evidence synthesis and structured dialogues, followed a standardized approach. This included (1) developing a policy brief and (2) producing a post‐dialogue summary. The management and coordination office disseminated these outputs through the SLHS platform's media channels, while the respective academic teams shared them within their learning communities.

## 3 Capacity Building and Training in the SLHS

To sustain and build the expertise needed to initiate LHS, the SLHS also integrated a strong focus on capacity‐building activities. A key component of the SLHS was research scientist training. In collaboration with the Swiss School of Public Health (SSPH+), a PhD Scholarship Program (PSP) trained doctoral researchers in LHS science and KT. Participants were expected to develop policy briefs, conduct dialogues and take courses in five core areas: (1) Design and Vision of a Learning Health System, (2) Health Systems and Services, (3) Evidence‐Informed Policy Framework, (4) Implementation Science, and (5) Communication in a Learning Health System [1].

These capacity‐building efforts also aligned with initiatives, like the Agency for Healthcare Research and Quality (AHRQ) and the Patient‐Centered Outcomes Research Institute programs in the United States, which train LHS scientists through mentored research scientist training embedded in health systems [15]. AHRQ has developed core competency domains, which guide training programs, including systems science, research methods, implementation science, and ethics [16, 17]. Similarly, Canada's CIHR Health System Impact Fellowship embeds doctoral and postdoctoral researchers in health systems, aiming to increase research capacity while equipping fellows with professional competencies relevant across micro to macro‐level health environments [18].

## 4 Research Aims and Objectives

While previous studies have explored several aspects of LHSs, there has been limited attention to national‐level implementation. This research aims to deepen understanding of the SLHS as a national LHS model and its contribution to evidence‐informed decision‐making (EIDM). Despite a growing body of work on academic policy engagement initiatives, they often lack sufficient detail. This study addresses that gap by examining the SLHS's role in informing EIDM in health policy and practice. This involves examining KT practices, methods and strategies that facilitate the ongoing integration of research into political processes, and KT activities, such as dialogues with learning communities. By uncovering lessons learned within the SLHS, the study seeks to identify recommendations that can inform institutionalizing KT efforts for EIDM in policy and sustaining the work of LHSs or similar platforms.

## 5 Research Questions

1. What are the individual experiences and perspectives of SLHS members, including senior academic professionals, current doctoral researchers, and alumni, involved in the platform's KT activities, and how do these experiences reflect the platform's impact and reach?
2. What challenges and successes have been encountered during the implementation of KT capacity building and practices by the SLHS?
3. What lessons from SLHS's KT activities and capacity building can guide future platform development, and what implications do these have for KT in evidence‐informed health policy?

## 6 Methods

A mixed‐methods approach, informed by Graham's [19] Knowledge to Action (K2A) framework, was used to assess the SLHS's role in KT for EIDM in policy, covering the period 2017–2023. The K2A framework was selected due to its alignment with the SLHS platform's established approach to KT. Since the platform already structures its activities around key K2A steps, this framework provided both a coherent methodological lens and a practical way to capture and analyze the data generated through the platform's work. Data sources included a document review, a descriptive survey, participatory workshops, and key informant interviews, providing both qualitative and quantitative insights.

### 6.1 Document Review

To assess SLHS's tangible outputs and reach, a document review was conducted. The review covered documents generated between SLHS's inception in 2017 and August 2023, including policy briefs, website analytics, and social media metrics.

Additionally, direct feedback from learning communities, collected through surveys and provided during and after dialogues, contributed to the review. The reviewed materials were categorized into three thematic areas in KT.

#### 6.1.1 KT Activities

To assess the diversity and degree of learning community participation through attendance lists, feedback forms, and surveys that were previously collected.

#### 6.1.2 KT Practices

To evaluate SLHS's role, including knowledge products and types that were produced, in informing health policy and practice decisions.

#### 6.1.3 KT Capacity Building

Focusing on the impact of workshops, training, courses, and events for SLHS members and health system professionals through feedback forms and surveys that were previously collected.

### 6.2 Survey

A descriptive survey explored SLHS members' perceptions of the platform's impact on KT and its relevance to EIDM. All members were invited to participate, which included senior academic professionals who were also governing board members, as well as current and former doctoral researchers of the SLHS. The survey, conducted from October to November 2023 via Qualtrics, sought to:

1. Assess members' views on the effectiveness of SLHS's KT practices, activities, and capacity building, specifically through the PSP.
2. Evaluate the extent to which SLHS's efforts to promote knowledge tailoring, transfer and application to relevant audiences, including the general public, patients and caregivers, professionals working in health policy and practice.

Survey items were developed based on the K2A framework, including Knowledge Creation, Knowledge Adaptation, Knowledge Transfer, and Knowledge Application [19]. The survey instrument, including all questions, is included in Appendix A: Supporting Information.

### 6.3 Workshops

Two participatory workshops were held in September 2023, each with a different member group: (1) SLHS senior academic professionals (in‐person), and (2) SLHS doctoral researchers (online). All current senior academic professionals and doctoral researchers affiliated with the SLHS were invited to attend the workshops. Workshops followed a World Café facilitation model [20, 21, 22], with multiple rounds of small group discussions focused on the following themes:

1. Key successes and challenges in SLHS KT activities and practices, including reflections on learning community engagement strategies.
2. Lessons learned from KT capacity‐building efforts.

Each discussion was guided by probing questions designed to elicit personal experiences and reflections on SLHS's impact on EIDM. Discussions were documented using flipcharts for in‐person workshops and Miro boards for virtual workshops. Workshop questions are detailed in Appendix B: Supporting Information.

### 6.4 Key Informant Interviews

Semi‐structured interviews were conducted from October–November 2023 with SLHS members, including senior academic professionals, current doctoral researchers, and alumni. Participants were recruited using a combination of purposive and quota sampling to ensure representation from each SLHS member group and to include only those with experience in an SLC. Questions drew on the SLHS aims [1], the K2A framework [19] and activities and outputs captured from other evaluations of KT platforms [23]. The interview guide consisted of three key sections: (1) SLHS Involvement, (2) SLHS Aims, Activities and Outputs, and (3) Final Reflection and focused on:

1. Understanding key successes and challenges in how the SLHS created and integrated knowledge into products to drive continuous health system improvement.
2. Exploring barriers and opportunities for KT in the Swiss health system context.
3. Identifying enabling conditions and difficulties to KT capacity building in Switzerland with probing questions included to prompt deeper insights into issue identification, academic‐community partnerships, participatory decision‐making, knowledge adaptation, and application.

All interviews were transcribed using Microsoft Word, and data was extracted based on participants' responses to the interview questions. Appendix C in the Supporting Information contains the full interview guide.

### 6.5 Data Analysis

Thematic coding of qualitative data from the document review, workshops, survey and interviews was conducted by two coders (N.G.M. and S.Z.). The analysis followed an iterative process of theme identification and validation, using both deductive (based on predefined thematic areas: KT Activities, KT Practices and KT Capacity Building) and inductive (emerging from the data) approaches [24, 25]. All quantitative survey data were analyzed descriptively using RStudio, and findings were triangulated with qualitative data to understand the SLHS's impact and reach, inform further development of the platform and identify potential implications for the global discourse on KT for EIDM.

## 7 Results

This section presents the findings from the study, beginning with general participation and engagement metrics, moving to specific KT activities, and their impact, and concluding with the overarching challenges and lessons learned in institutionalizing KT practices.

### 7.1 Participation

#### 7.1.1 Survey

Among 34 respondents, 50% were men, 47.1% women, and 2.9% preferred not to disclose. Most were younger, with 41.2% aged 25–34 and 25.0% aged 35–44. In terms of professional roles, 41.2% were doctoral researchers, 32.4% senior academic professionals, and 8.8% program managers, senior scientists, or post‐doctoral researchers, showing academic and professional involvement at the alumni level of the program. The survey showed that a significant portion of respondents were actively engaged in key SLHS activities, with 70.6% contributing to policy briefs, and 73.5% participating in dialogues.

#### 7.1.2 Workshops

The first workshop was held with 22 participants representing senior academic professionals from 9 SLHS partner institutions; while the second workshop included 13 current doctoral researchers representing 8 SLHS partner institutions.

#### 7.1.3 Interviews

Ten interviews were conducted with SLHS members representing 8 SLHS partner institutions. This included two current doctoral researchers, two alumni, and six senior academic professionals affiliated with the SLHS.

### 7.2 KT Activities and Practices

#### 7.2.1 Learning Community Engagement and Participatory Decision‐Making

The document analysis showed that since its launch in 2017, the SLHS has consistently demonstrated strong engagement efforts through its KT activities. Between 2017 and August 2023, over 235 learning community members participated in 26 dialogues, each informed by one of 19 policy briefs.

To further explore SLHS engagement, the interviews and workshops gathered SLHS members' perspectives, while evaluation forms from past dialogues captured insights from learning community members. Feedback was largely positive, with many expressing openness to further involvement. Interview participants shared their experiences with organizing and conducting dialogues, discussing different formats, including length, participant diversity, or methodology that were used and could be considered in the Swiss context. Most stated that the choice of dialogue format is closely tied to the topic of each SLC. Its sensitivity and perceived feasibility, especially from the perspective of community partners involved, were important considerations in these decisions.

67.5% (*N* = 23) of survey participants agreed that the SLHS effectively established a bridging mechanism between research, policy, and practice by fostering participatory decision‐making through dialogues. Some respondents suggested expanding engagement, especially with patients, and integrating it more routinely and systematically within the health system. One respondent mentioned forming an advisory council to better involve community partners in research, while another suggested considering ways to professionalize their engagement.

#### 7.2.2 Adapting KT Strategies for Impact Across Contexts

Evaluation forms from past dialogues highlighted the effectiveness of the SLHS's KT practices and activities, particularly how policy briefs and dialogues have facilitated the adaptation and transfer of research knowledge, reflecting the platform's strategic approach to EIDM in policy. In 2023, feedback from a series of five dialogues showed that 81.7% (49 out of 60) of participants had an overall positive experience, and 80% (48 out of 60) felt they had sufficient information to engage effectively in the discussions.

**FIGURE 3:**
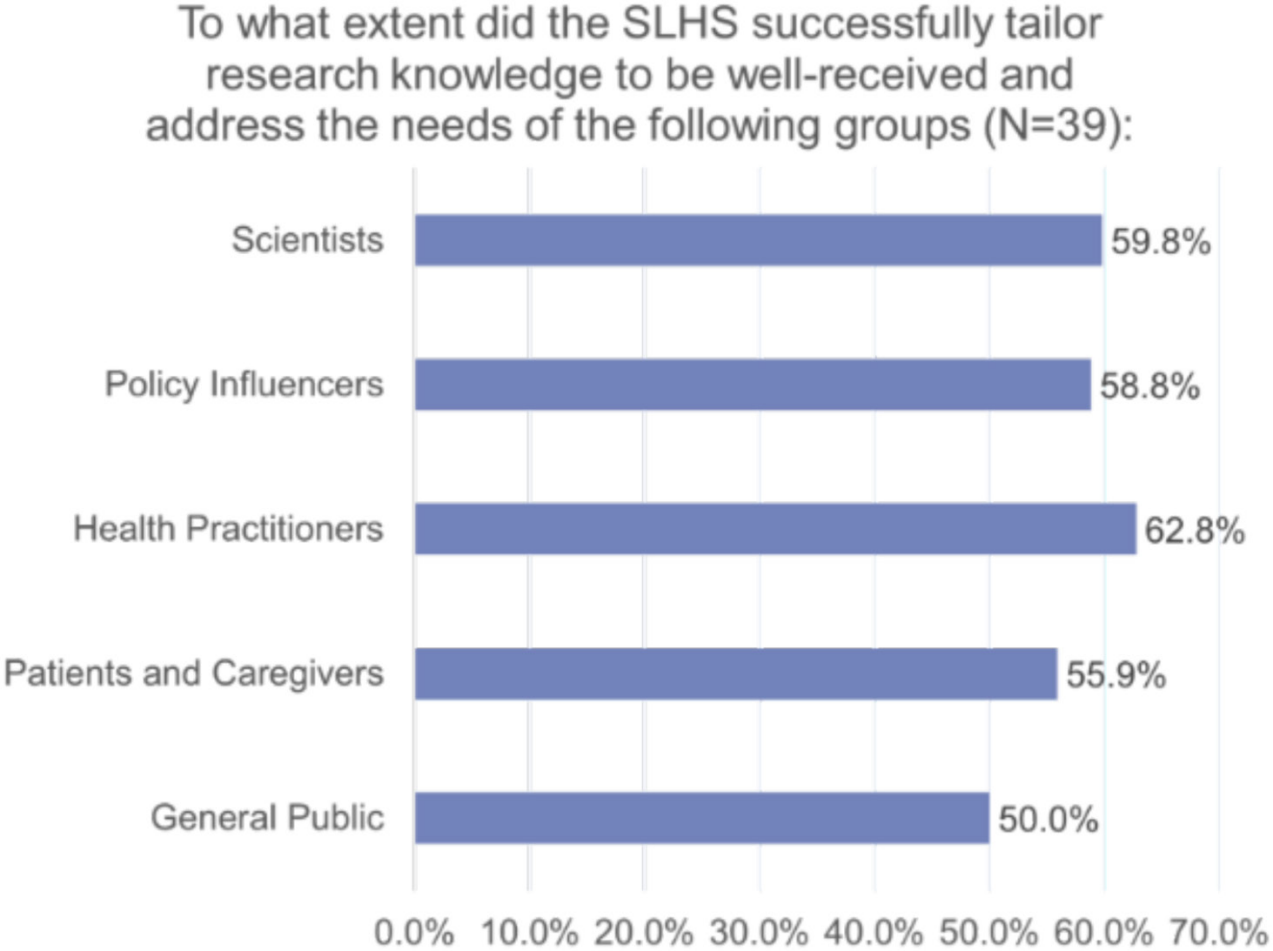
To what extent did the SLHS successfully tailor research knowledge to be well‐received and address the needs of groups within learning communities.

In the survey with SLHS members only, participants were asked about the platform's capability to adapt research findings to knowledge products relevant to local contexts. This question considered factors such as the usefulness, timeliness, and appropriateness of knowledge products, such as policy and evidence briefs, to their specific setting and situation. Only half of the respondents believed the SLHS effectively adapted research knowledge for the general public. Additionally, they reported successful tailoring for other groups: 55.88% for patients and caregivers, 62.75% for healthcare practitioners, 58.82% for policy influencers, and 59.80% for the scientific community (Figure 3).

The survey also questioned the SLHS's ability to transfer research knowledge, a deliberate process of disseminating knowledge products aimed at increasing its usage. Nearly three‐quarters of respondents believed the platform effectively transferred research knowledge to healthcare practitioners (73.5%), policy influencers (76.5%), and scientists (73.6%). However, engagement was perceived as less effective among community members, including the general public and patients and caregivers, with 47.3% and 41.2% reporting effective knowledge transfer.

#### 7.2.3 Challenges in Policy Influence and Decision‐Making

While many successes were discussed, translating research into actionable policy changes remained challenging for workshop participants and interviewees. Workshop participants attributed these difficulties to the lack of apparent interest from decision‐makers and the protracted nature of political processes. Interviewees expressed that they were unaware of the dissemination process for policy and evidence briefs produced, as well as the summaries of the dialogues. These factors contributed to SLHS members' uncertainty in assessing the influence of knowledge products on decision‐making, with only 44.1% of survey respondents believing that those working in health policy incorporate or apply the research knowledge or evidence produced by the SLHS in their decision‐making processes. This was also partly reflected in the dialogues, as participants noted challenges in the clarity of communication from SLHS members and the dissemination of dialogue summaries.

**FIGURE 4:**
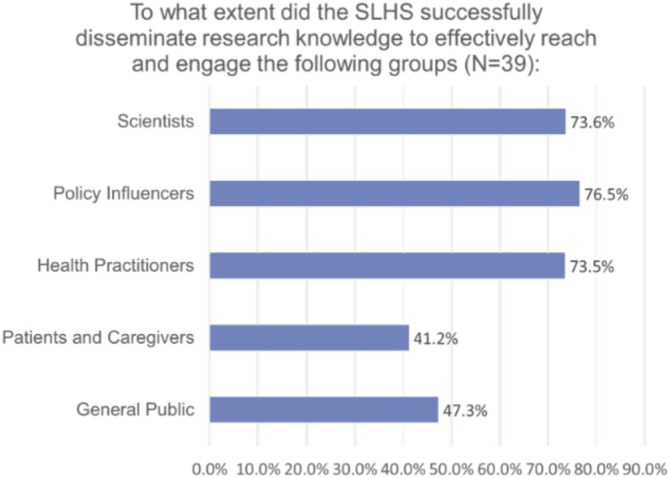
To what extent did the SLHS successfully disseminate research knowledge to effectively reach groups within learning communities.

To further assess SLHS KT activities in promoting EIDM in policy and practice, interviews and workshops also gathered SLHS members' perspectives, which highlighted another challenge: not all community partners readily embrace traditional academic research or policy briefs. This supports the survey results above, which show that SLHS members feel less effective in transferring research knowledge to the general public, patients, and caregivers (see Figure 4).

#### 7.2.4 Strengthening KT Through Strategic Co‐Creation

Workshop and dialogue participants, as well as interviewees, emphasized the importance of ongoing engagement and working closely with a broader range of health system decision‐makers for improving the routine use of evidence. Many advocated for a participatory approach, where researchers in partnership with learning communities, including policymakers, patients, social services and healthcare providers from the beginning to develop priorities, knowledge, and solutions.

Workshop participants and interviewees identified co‐creation as a potential strategy to tailor knowledge products more effectively to meet audience‐specific needs. They further discussed that this could aid in ensuring the collaborative development of knowledge products, potentially enhancing their relevance and impact by accommodating diverse requirements, such as language proficiency, competency levels, and cultural nuances. In addition, participants in the dialogues stressed that training health system professionals to engage meaningfully in learning communities, along with sufficient time for informal relationship‐building, would facilitate the adoption of the platform and its practices.

### 7.3 Capacity Building for KT

#### 7.3.1 Engaging Health System Professionals and Research Scientists

The document review highlighted capacity building as a core SLHS activity, with almost 120 health system professionals trained, 35 knowledge exchange activities conducted (e.g., conferences, webinars), and 50 doctoral researchers (29 current, 21 alumni) engaged across thematic areas. In the survey, 82.35% of respondents expressed that the SLHS effectively fosters a bridging mechanism between science, policy, and practice by enhancing the capacity of available research scientists through the PSP. Workshop participants and interviewees emphasized the value of health systems and services research training, noting its important role in enabling researchers to operate at the interface of science, policy, and practice. They highlighted that the integration of theoretical KT training with practical application, especially through dialogues and policy briefs, was particularly effective.

#### 7.3.2 Structural Challenges in Scaling KT Efforts

While capacity‐building efforts were widely praised, participants noted challenges in institutionalizing KT and ensuring long‐term sustainability required for routine EIDM in policy. Relying solely on the capacity of doctoral researchers and senior academic professionals in addition to a small management and coordination office limits the model's capacity. The typical three–to–four‐year duration of doctoral projects, along with expectations for significant academic output and learning community engagement activities, such as policy briefs and dialogues, places substantial demands on resources.

Moreover, workshop participants and interviewees expressed concerns about the human capital required to meet the initiative's aim of customizing knowledge products to diverse audience needs, especially patients and caregivers, healthcare practitioners, and the general public. They also highlighted the challenge of maintaining learning community relationships through continuous capacity building, communication, and shared decision‐making. Another recurring theme in the interviews was the loss of knowledge at the end of doctoral projects, often resulting in the need to restart efforts rather than build on previous work and monitor and/or follow up on policy briefs and dialogues. This challenge is compounded by the fact that current doctoral funding periods are often shorter than political processes, limiting the potential for sustained EIDM impact on health policy.

#### 7.3.3 Building Sustainable Capacity for KT and EIDM

Despite the challenges outlined above, the initiative was commended for fostering a culture shift that facilitated community building, collaboration and breaking down silos in the Swiss health system. A key lesson was the need for more flexible funding mechanisms to ensure the long‐term sustainability and institutionalization of KT. Participants also noted the need for additional human resources to develop tailored knowledge products and meet learning community needs, as well as the potential to affect health system improvement.

Furthermore, a key lesson from the workshops was the importance of providing comprehensive guidance and mentorship to doctoral researchers, aligning their research, policy briefs, and dialogues with relevant health system issues. While senior academic professionals provided significant support, there is a need for more systematic integration of community knowledge and health system professionals' experiences into academic research and training.

## 8 Discussion

The study's findings highlight the role of KT in strengthening EIDM through a LHS approach, emphasizing the need for KT strategies to be carefully adapted to different decision‐making environments. While a top‐down structure like the SLHS promotes a unified approach, Schleyer et al. [5] note that it also demands flexibility to address cultural and scalability challenges. The study's findings support this and highlight the importance of co‐creation practices that are adaptable across contexts. KT products, such as policy briefs, must include diverse knowledge sources, especially often‐excluded voices, such as patients and caregivers.

Our study also points to gaps in current top‐down dissemination strategies. These often overlook key community partners, limiting their ability to foster context‐specific knowledge mobilization. The findings emphasize the necessity of routine capacity building and ongoing engagement with learning communities. Infrequent interactions tend to diminish the opportunities for co‐production and potentially the uptake of evidence in decision‐making. Additionally, incorporating lived experiences into research training emerges as a pivotal strategy for grounding research in practical real‐world applications; therefore increasing the potential relevance and effectiveness of health system improvements.

### 8.1 Challenges in Knowledge Tailoring and Transfer

The findings confirm that the SLHS served as a key conduit between science, policy, and practice; however, even with substantial capacity‐building efforts, there is still a need for more tailored approaches to knowledge adaptation and dissemination. While Geese and Schmidt [26] suggest that the SLHS holds potential for improving health system challenges, the findings demonstrate that the impact of its KT efforts can be easily stifled by insufficiently tailored KT efforts and limited resources to meet communities' needs for engagement in its operations. To further improve KT efforts, the findings stress the need for co‐creation strategies and the extension of community‐led transformation principles (CLT), which include involving the learning community not only in priority setting and solution development but also actively in tailoring knowledge products. In addition, co‐developing targeted dissemination strategies with learning communities, using local networks and context‐specific channels, could also be an opportunity to improve the effectiveness of KT efforts for EIDM. Furthermore, these approaches may enhance context‐specific practices in research and foster a more equitable distribution of power among partners [27].

Despite successes in learning community engagement and knowledge co‐creation, the study identified challenges in the SLHS platform's ability to influence policy. Addressing issues such as engaging decision‐makers and translating knowledge requires creating varied formats of products and activities and using accessible channels to ensure broader reach and applicability. The Translation‐to‐Policy Learning Cycle (T2PLC), as discussed by Kilbourne et al. [28], provides a relevant framework for tackling these specific issues. The T2PLC is described as a promising framework that centers co‐creation and extends previous LHS versions to address these challenges in policy influence and health system improvement [28, 29].

### 8.2 Extending Learning Community Engagement and Capacity Building

The study indicated that the largest success of the SLHS was its capacity‐building efforts for health system professionals and doctoral research scientists' training. Findings suggest that these efforts may have initiated a cultural shift influencing the health system's adaptability and responsiveness by training scientists and professionals to think across the intersections of policy, practice, and science. Furthermore, the study emphasized that this approach helped break down silos and fostered community building through participatory methods. Aligning with the outcomes of LHS training programs in Canada and in the USA, training a workforce in LHS competencies is regarded as potentially enhancing health system resilience and generating a significant potential for systemic impact [15, 30].

### 8.3 Limitations and Future Direction

While this study offers an analysis of the SLHS's efforts, its sample size and focus on SLHS members and feedback forms from dialogue participants may limit the generalizability of the results. As a result of this work, future research should aim to incorporate perceptions and experiences from a wider array of engaged community partners and knowledge users to enhance the representativeness of the findings. One limitation of this work is that no explicit funding was dedicated to support the development and sustainment of learning communities or the reach of their work. This raises questions about their long‐term viability, making it important to explore the longitudinal effects of community engagement on the uptake of evidence in policy and practice, which could provide important perspectives on the sustainability and impact of these efforts.

In conclusion, this study highlights the key role of IKT approaches, such as the SLHS framework. By promoting inclusivity and CLT principles, the SLHS has the potential to enhance its impact on health system improvement and policy development. These findings provide not only a theoretical contribution to the field of KT and LHS, but also practical guidance for health systems aiming to integrate community knowledge into their decision‐making processes.

## Funding

This work was supported by the Swiss State Secretariat for Education, Research and Innovation.

## Conflicts of Interest

The authors declare no conflicts of interest.

## Supporting information


**Data S1:** Supporting Information.

## Data Availability

Research data are not shared.
