# Supplementary material for: Integrating Knowledge Translation: A Swiss Approach to Bridging Research and Health System Improvement
Source: Learn Health Syst. 2025 Dec 5;10(1):e70056. doi: 10.1002/lrh2.70056 (PMC12805882; doi:10.1002/lrh2.70056)
Supplement: Supplementary file 1 — Data S1: Supporting Information. [file LRH2-10-e70056-s001.docx]

Appendix A. (Survey)

| **Section 1: Background Information** |
| --- |
| 1. Age: [18 to 24/25 to 34/35 to 44/45 to 54/55 to 64/65 and over] |
| 2. Gender: [Man/Non-binary/Woman/Prefer not to say/Prefer to self-describe:] |
| 3. Current role: [Doctoral researcher, Post-doctoral researcher, Professor (Assistant, Associate or Full), Senior scientist, Other:] |
| 4. In your research or professional involvement with the SLHS, which specific geographical area(s) have been your primary focus? [French-speaking region/German-speaking region/Italian-speaking region/Romansh-speaking region] |
| 5. What year did you join the SLHS? [2017, 2018, 2019, 2020, 2021, 2022, 2023] |
| Since joining the SLHS, have you developed a policy brief? [Yes/No/Not yet/Currently in the process of developing a policy brief] |
| Since joining the SLHS, have you participated in the development of a structured dialogue? [Yes/No/Not yet/Currently in the process of organizing a structured dialogue] |
| How many SLHS Annual & Mid- Term Meetings have you attended? [1-3/4-6/7 or more/None] |
| **Section 2: SLHS Aims (Boes et al, 2018)** |
| **1. Bridging Mechanism:** |
| Since its inception, the SLHS effectively established a bridging mechanism between research, policy, and practice: |
| 1a. Through a systematic and ongoing dialogue between researchers, policymakers, and practitioners, the SLHS is continuously **identifying and monitoring current challenges (issues) in the Swiss health system**. (Please state your level of agreement below) |
| Response options: Strongly disagree to Strongly agree and Unsure |
| 1b. Do you have any ideas or suggestions for refining and strengthening this component of the SLHS? [Open-ended response] |
| 2a. With academic partner institutions and their networks actively **contributing to evidence syntheses and creating policy briefs for structured dialogues** to strengthen the connection between research and practical application. (Please state your level of agreement below) |
| Response options: Strongly disagree to Strongly agree and Unsure |
| 2b. Do you have any ideas or suggestions for refining and strengthening this component of the SLHS? [Open-ended response] |
| 3a. By **fostering participatory decision-making in health policy at structured dialogues**. This process involves participants interacting to identify and reach consensus on optimal solutions for addressing specific problems and issues. (Please state your level of agreement below) |
| Response options: Strongly disagree to Strongly agree and Unsure |
| 3b. Do you have any ideas or suggestions for refining and strengthening this component of the SLHS? [Open-ended response] |
| 4a. Through **enhancing the capacity of available research scientists through the SLHS-SSPH+ Doctoral Scholarship Program**. This program equips scholars with the skills to understand and structure research at the intersection of science, policy, and practice. (Please state your level of agreement below) |
| Response options: Strongly disagree to Strongly agree and Unsure |
| 4b. Do you have any ideas or suggestions for refining and strengthening this component of the SLHS? [Open-ended response] |
| **Section 3: Knowledge to Action Framework (Graham et al, 2006) & (Partridge et al, 2020)** |
| **1. Knowledge Creation:** |
| The SLHS effectively contributed to evidence syntheses by producing: (Please state your level of agreement below) |
| 1. Policy Briefs |
| 2. Systematic & Scoping Reviews |
| 3. Traditional Research Outputs (e.g. original research in peer-reviewed journal articles) |
| Response options: Strongly disagree to Strongly agree and Unsure |
| 4. Do you have any ideas or suggestions for refining and strengthening evidence syntheses of the SLHS? [Open-ended response] |
| **2. Knowledge Adaptation:** |
| 1. To what extent did the SLHS successfully tailor research knowledge to be well-received and address the needs of the following groups: [Add Definitions of Each Group] |
| a. General Public: For example, by translating complex research findings into easily understandable materials. |
| b. Patients and Caregivers: Customizing research insights into relevant, practical and easily understandable materials. |
| c. Healthcare Practitioners: For instance, by providing study outcomes in a manner that could improve patient care practices. |
| d. Policy Influencers: Such as providing data-driven insights to inform healthcare policy and funding decisions or guide legislative actions. |
| e. Scientific Community: Including published research findings in peer-reviewed journals. |
| Response options: To a very great extent to Not at all and Unsure |
| 2. Additionally, do you have any ideas or suggestions for enhancing the tailoring of SLHS research knowledge to better meet the needs of these groups? |
| **3. Knowledge Transfer:** |
| 1. To what extent did the SLHS successfully disseminate research knowledge to effectively reach and engage the following groups: |
| a. General Public: This could include efforts such as structured dialogues, creating accessible informational materials, organizing events, or utilizing digital media. |
| b. Patients and Caregivers: This may involve structured dialogues, targeted communication campaigns and partnerships with relevant organizations to increase the reach findings. |
| c. Healthcare Practitioners: This may encompass strategies such as structured dialogues, disseminating research products to professional societies, developing capacity building or "research to practice" workshops, or online resources for practitioners. |
| d. Policy Influencers: This involves initiatives like distributing policy briefs to political actors, structured dialogues, presenting research findings at policy forums, developing capacity building or "research to policy" workshops, or actively collaborating with policymakers. |
| e. Scientific Community: This could include activities such as structured dialogues, presenting research at academic conferences, collaborating with researchers from partner institutions, or contributing to the scientific discourse through workshops and seminars. |
| Response options: To a very great extent to Not at all and Unsure |
| 2. Additionally, do you have any ideas or suggestions for improving the dissemination efforts of the SLHS? [Open-ended response |
| **4. Knowledge Application:** |
| 1. To what extent do you perceive the following groups incorporate or apply the research knowledge or evidence produced by the SLHS in their decision-making processes or practices? |
| a. Healthcare Practitioners |
| b. Policy Influencers |
| c. Scientific Community |
| Response options: To a very great extent to Not at all and Unsure |
| 2. Additionally, do you have any ideas or suggestions for enhancing the application or influence of SLHS research knowledge or evidence within these groups?[Open-ended response] |
| **5. Knowledge Evaluation:** |
| 1. The SLHS is committed to evaluating its impact and the effectiveness of research knowledge in bringing about health system changes. |
| Response options: Strongly disagree to Strongly agree and Unsure |
| 2. How would you rate the feasibility of evaluating the SLHS in terms of its impact and effectiveness? |
| Response options: Highly feasible to Highly challenging and Unsure |
| 3. Do you have any ideas or suggestions for refining and strengthening evaluation efforts of the SLHS? [Open-ended response] |
| **Section 4: Final Remarks** |
| 1. If you have any further comments or would like to share additional insights, please feel free to do so in the space provided below. [Open-ended response] |

Appendix B. (In-Person & Online Workshop Questions)

World Café Round I Question: "What are the key successes and challenges of the SLHS?"

Probing Questions:

- *In your experience with the SLHS, what are some of the strengths in terms of engaging community partners, the dialogues and other activities? And what are some of the weaknesses?*
- *Can you share any success stories related to the policy briefs, dialogues and other activities by the SLHS? Can you elaborate on some of the challenges?*
- *In your opinion, what are some of the advantages within the SLHS regarding promoting interdisciplinary collaboration or usage of the policy? And what about some of the difficulties?*

World Café Round II Question: "What are the key successes and challenges of the SLHS?"

Probing Questions:

- *In your experience with the SLHS, what are some of the strengths in terms of engaging community partners, the dialogues and other activities? And what are some of the weaknesses?*
- *Can you share any success stories related to the policy briefs, dialogues and other activities by the SLHS? Can you elaborate on some of the challenges?*
- *In your opinion, what are some of the advantages within the SLHS regarding promoting interdisciplinary collaboration or usage of the policy? And what about some of the difficulties?*

World Café Round III Question: "What are the key lessons learned in the SLHS?"

Probing Questions:

- *Reflecting on your experience with the SLHS, what valuable lessons have you learned along the way?*
- *Can you share any specific insights or lessons you gained from your SLHS involvement?*
- *Thinking back on your journey within the SLHS, what lessons have you personally taken away that have influenced your understanding or approach to knowledge translation?*
- *What experiences or insights have influenced your understanding of how science, policy, and practice interact and influence each other?*
- *Considering the challenges and successes you have encountered within the SLHS, what lessons or insights would you like to share with others who may be embarking on a similar initiative?*

Appendix C. (Interview Questions)

Section 1:

SLHS Involvement

1. Can you describe your role and responsibilities within the SLHS?

Probing questions:

- Which regions within Switzerland does your work focus on?
- What type of non-academic partners do you typically collaborate with?

2. What motivated you to be a part of the SLHS, and how has your involvement evolved since you joined?

3. Do you have experience developing, contributing to, or supervising the creation of policy briefs and structured dialogues within the SLHS?

Probing questions:

- What worked well during the process?
- What challenges did you all encounter?
- Were there any interdisciplinary collaborations or external partnerships in creating the policy briefs? If so, how did they influence the process?
- Were there any resources or tools that you found particularly useful during the creation of the policy briefs?

4. Regarding SLHS meetings, such as the annual and mid-term meetings, are you attending those regularly?

Follow-up & probing questions:

- What are often the key takeaways from these meetings?
- What value do these meetings have for the SLHS partners involved?
- What other productive activities could we engage in as a network to improve the functioning of the SLHS?

Section 2:

SLHS Aims (Boes et al, 2018) & The Knowledge to Action Framework (Graham et al, 2006 & Partridge et al, 2020**)**

1. **SLHS Aim I: Issue Identification & Issue Monitoring (Boes et al, 2018):**

In your experience, how well did the SLHS in identifying and monitoring current issues in the Swiss health system by the SLHS?

Probing questions:

- Can you share an example of a specific achievement or barrier in the Swiss health system that the SLHS has identified?
- How feasible is it for the SLHS to monitor health challenges in the Swiss health system?
- What are facilitators and what are barriers to the identification of issues?
- Do you have any suggestions on how to improve issue identification?

1. **SLHS Aim II: Academic Partnerships and Evidence Syntheses (Boes et al, 2018):**

How well have the SLHS’ (this includes academic partner institutions and their networks) processes actively contributed to evidence syntheses (such as policy briefs, systematic and scoping reviews)?

Probing question:

- Are there specific strategies or practices you believe have been particularly effective in producing knowledge?
- How, if related, has the SLHS partnerships fostered collaboration for evidence syntheses?
- Could you describe the collaboration and coordination between researchers, policymakers, and practitioners when producing policy briefs and other knowledge products within the SLHS?

1. **Knowledge Creation (Graham et al, 2006 & Partridge et al, 2020):**

Now talking about impact, in your opinion, what has been the impact of the SLHS in terms of producing policy briefs, systematic & scoping reviews, and traditional research outputs?

Probing questions:

- How relevant do you find the knowledge that is produced?
- In your opinion, what could be done to enhance the impact and reach of the SLHS's knowledge production efforts, especially in terms of policy briefs and systematic/scoping reviews?

1. **Knowledge Adaptation (Graham et al, 2006 & Partridge et al, 2020):**

Can you provide insights and specific examples of how the SLHS has effectively tailored research knowledge to various groups, including the general public, patients, caregivers, healthcare practitioners, policy influencers, and researchers?

Probing questions:

- What are the key challenges and opportunities encountered when tailoring research knowledge to diverse groups, and from your experience, how does the SLHS navigate them?
- How does the SLHS ensure that research findings are presented in a way that is easily understandable, relevant, and accessible to different groups?
- In your opinion, what could be done to enhance the adaptation of SLHS research knowledge to better meet the needs of these diverse groups?

1. **Aim III: Participatory Decision-Making & Co-creation (Boes et al, 2018):**

How well did the SLHS successfully facilitate dialogue participants' interaction to identify and reach consensus on solutions for addressing a specific health system problem or issue?

Probing questions:

- What were the outcomes of this process?
- What role do you believe participatory decision-making and co-creation plays in the effectiveness of the SLHS's initiatives? Are there areas where you see opportunities for improvement?

1. **Knowledge Transfer (Graham et al, 2006 & Partridge et al, 2020):**

When it comes to disseminating the research knowledge produced by the SLHS, how do you perceive the success of the SLHS in reaching and engaging the various groups previously mentioned?

Probing questions:

- In your view, what specific strategies or methods have proven to be the most effective in reaching and engaging the diverse groups mentioned earlier?
- Are there any challenges or barriers that the SLHS encounters in disseminating research knowledge to these various groups, and how does the organization address these challenges?
- In your opinion, what additional measures or approaches could be implemented to enhance the success of knowledge dissemination to diverse groups?

1. **Knowledge Application (Graham et al, 2006 & Partridge et al, 2020):**

Regarding the application of research knowledge produced by the SLHS, can you share your perspective on the extent to which healthcare practitioners, policy influencers, and researchers incorporate or apply this knowledge in their decision-making processes or practices?

Probing questions:

- Are there ongoing collaborations or mechanisms in place that encourage policy influencers/health practitioners/researchers to seek and utilize SLHS research in their work?
- In your view, how receptive have healthcare practitioners/policy influencers/researchers been to applying research knowledge from the SLHS in their clinical practices?
- Are there any suggestions or ideas you can offer to increase the application or influence of SLHS research knowledge within these groups?
- What are the key factors or barriers that influence the successful application of SLHS research knowledge by different groups, and how does the SLHS address these factors?

1. **Aim IV: SLHS-SSPH+ Doctoral Scholarship Program (Boes et al, 2018):**

From your perspective, can you describe the impact of the SLHS-SSPH+ Doctoral Scholarship Program on equipping research scientists with the skills to understand and structure research at the intersection of science, policy, and practice?

Probing questions:

- Do you have any suggestions for enhancing the capacity-building aspect of the SLHS-SSPH+ Doctoral Scholarship Program?
- Are there any components missing in the scholarship program?
- What about any thoughts for expanding the program's reach and impact?

1. **Knowledge Evaluation (Graham et al, 2006 & Partridge et al, 2020):**

What are your thoughts on the SLHS's commitment to assessing its impact and the effectiveness of its research knowledge in driving change within the healthcare system?

Probing questions:

- In your experience, what challenges or barriers does the SLHS encounter when evaluating the impact of its research knowledge?
- Are there any noteworthy success stories linked to the evaluation or existence of the SLHS?

Considering the feasibility of evaluating the SLHS's impact and effectiveness, what factors do you believe make it more or less challenging?

Probing questions:

- What steps can we take to improve the evaluation process?
- In your opinion, how does the availability of resources, such as funding and personnel, affect the SLHS's ability to conduct comprehensive impact evaluations?
- Considering the complexity of the Swiss healthcare system, what aspects of this complexity pose challenges to effectively evaluating the SLHS's impact and effectiveness?
- How does the time frame for observing the impact of research knowledge produced by the SLHS affect the feasibility of evaluation?
- In your view, is it necessary for the SLHS to engage in benchmarking or comparative analysis with other learning health systems or knowledge translation initiatives to assess its impact?

1. **General Reflection on SLHS Aims:**

Since we just talked about the SLHS aims and, for a quick reflection, how would you assess the overall effectiveness of the SLHS in achieving its aims?

Probing questions:

- Are there any specific areas within the aims of the SLHS that you believe require further attention or improvement?
- Can you provide specific examples that demonstrate the SLHS's success in achieving its aims?

Section 3:

Final Remarks

As our discussion draws close, I greatly appreciate your valuable perspective. Before we conclude, I'd like to offer you the opportunity to share any last remarks or thoughts you believe should be included.

1. Is there anything else you would like to add or any additional insights you'd like to share about your experience with the SLHS, its aims, or its activities?
